# A MicroRNA Derived From *Schistosoma japonicum* Promotes Schistosomiasis Hepatic Fibrosis by Targeting Host Secreted Frizzled-Related Protein 1

**DOI:** 10.3389/fcimb.2020.00101

**Published:** 2020-03-13

**Authors:** Yange Wang, Xiaobin Fan, Nanhang Lei, Xing He, Xiaoxi Wang, Xufeng Luo, Dongmei Zhang, Weiqing Pan

**Affiliations:** Department of Tropical Diseases, Naval Medical University, Shanghai, China

**Keywords:** *Schistosoma japonicum*, microRNA, hepatic fibrosis, SFRP1, cross-species regulation

## Abstract

Schistosomiasis remains a serious parasitic disease, which is characterized by granulomatous inflammation and hepatic fibrosis. MicroRNAs derived from parasites can regulate host genes and cell phenotype. Here, we showed that a miRNA derived from *S. japonicum* (Sja-miR-1) exists in the hepatic stellate cells (HSCs) of mice infected with the parasite and up-regulates the expression of collagens and α-SMA by targeting secreted frizzled-related protein 1 (SFRP1). A vector-mediated delivery of Sja-miR-1 into naive mice led to hepatic fibrogenesis in the mice. Accordingly, inhibition of Sja-miR-1 in the infected mice led to reduction of the parasite-induced hepatic fibrosis. The mechanism behind the Sja-miR-1-mediated activation of HSC could be through targeting SFRP1 to regulate the Wnt/β-catenin pathway. These findings reveal that parasite-derived small non-coding RNAs are implicated in cross-species regulation of host pathological process and persistent inhibition of Sja-miR-1 may provide a therapeutic potential for the parasite diseases.

## Introduction

At present, schistosomiasis is still a serious parasitic disease, which infects more than 240 million people across 78 countries (Colley et al., 2014). *Schistosoma mansoni* and *Schistosoma japonicum* are the major species that cause liver disease in humans. Followed by infecting humans or animals, schistosome cercariae migrate to the host portal-mesenteric vein system where the female worm lay a large number of eggs that, at a part, are transported to the liver through portal veins, causing egg granuloma and hepatic fibrosis, eventually form cirrhosis which is the main cause of mortality of schistosomiasis.

Schistosomiasis is actually an immune pathological disease (Pearce and Macdonald, 2002). At the early stage of schistosome infection, Th1 immune response characterized by elevated IFN-γ is moderately activated. When the eggs deposit in the tissue, the immune response is quickly polarized to Th2 response marked by elevation of IL-13 and IL-4 (Pearce and Macdonald, 2002; Wilson et al., 2007). The Th2-related cytokines are important mediators for regulation of schistosomiasis hepatic fibrosis and progression of the disease (Wilson et al., 2007). Hepatic HSCs have been proven to be the main effector cells of the hepatic fibrosis (Bartley et al., 2006). The eggs trapped in liver induce activation of quiescent HSCs to the proliferative and fibrogenic myofibroblasts, which generate excessive extracellular matrix (ECM), leading to hepatic fibrosis (Burke et al., 2009). The inhibition of the activated HSCs provides a possibility of developing tools for prevention and treatment of the fibrosis (Friedman, 2008).

MicroRNAs (miRNAs), a highly-conserved and endogenous non-coding RNA, widely exist in animals and plants that regulate post-transcriptional gene expression and play an important role in progression of many diseases (Bartel, 2004; Lu et al., 2008). Some miRNAs of plants and parasites have been found in human or animal body fluids and even entry into host cells to modulate host genes and their phenotype in a cross-species manner (Zhang et al., 2012; Hu et al., 2019). MiR-168a abundant in rice can regulate the expression of the gene encoding low-density lipoprotein receptor adapter protein 1 in mammals (Zhang et al., 2012). Small RNAs of fungal, can silence plant genes related to host immunity (Wang et al., 2016). Parasite-derived miRNAs can be absorbed by the intestinal epithelial cells in mice and inhibit host type 2 innate immunity (Buck et al., 2014). Recently, several miRNAs of *Schistosoma japonicum* were reported to be involved in the modulation of host macrophage proliferation and pathogenesis of schistosomiasis by targeting relative genes of host (He et al., 2019; Liu et al., 2019).

In schistosomiasis, a granulomatous reaction occurs resulting from eggs trapped in the liver tissues, surrounded with immunocytes and other hepatic mesenchymal cells including HSCs (Wilson et al., 2007; Anthony et al., 2012). Exosomes are well-verified to play an important role in pathogen-host interaction or cell-cell communication. Recent studies showed that *S. japonicum* eggs could release exosomes containing miRNAs that could be transported into recipient cells (Zhu L. et al., 2016; Zhu S. et al., 2016). In this study, we showed a miRNA derived from *Schistosoma japonicum* exists in HSCs of mice infected with the parasite and contributed to the HSCs activation resulting in hepatic fibrosis. We demonstrate that the miRNA exerts the fibrosis-promoting effect through targeting the gene encoding SFRP1 of the host.

## Materials and Methods

### Ethics Statement

All the animal experiments were conducted consistent with the Guidelines of the Care and Use of Laboratory Animals of the National Institutes of Health, and the study received the approval of the Animal Ethics Committee of Naval Medical University (access number FYXK [Shanghai] 2014-0003).

### Mice and Parasite Infections

Six-week-old male BALB/c mice which were all bought from the experimental animal center of the Naval Medical University (Shanghai, China) were raised under a specific pathogen-free conditions, padded, controlled temperature (23 ± 2°C) and photoperiods (1:1 light-dark cycle). In order to establish a schistosomiasis mouse model, mice were percutaneously exposed to 16 *S. japonicum* cercariae shed from the lab-cultured snails (*Oncomelania hupensis*) obtained from the National Institute of Parasitic Disease, Chinese Center for Disease Control and Prevention (Shanghai, China).

### RNA Preparation and Quantitative Real-Time PCR (qRT-PCR)

Total RNA was extracted from the liver tissue or isolated HSCs with the Trizol reagent (Sigma-Aldrich, #T9424) and specific procedure was performed according to manufacturer's protocol. The expression of sja-miR-1, *Col1*α*1, Col3*α*1*, α*-Sma, Sfrp1* were detected with a SYBR Green Master Mix kit (Roche, #04913914001). U6 snRNA and β*-Actin* were used as endogenous controls to normalize the real-time PCR data, and the relative expression was calculated by the 2^−ΔΔCt^ method. The primers used in this study are shown in Table S1.

### Isolation and Culture of Primary HSCs

We isolated primary HSCs from mouse liver using the previous protocol (He et al., 2015). Briefly, the livers were initially perfused with pronase/collagenase type IV through the hepatic portal vein, and then the digested liver tissues were triturated and suspended in cold DMEM with 4% fetal bovine serum and antibiotics under a sterilized condition. The hepatocytes were isolated from the collected cell suspension by two runs of centrifugation at 50 g for 4 min each and hepatocytes were pelleted. Then the remaining supernatant was centrifuged at 500 g for 5 min to collect the non-parenchymal cells. Further, the sediment was resuspended in DMEM and added to 11.5% iodixanol gradient (Axis-Shield, #AS1114542) by centrifugation at 1,400 g for 20 min. The HSCs that enriched at the interface of iodixanol gradient and DMEM were resuspended in PBS and centrifugated at 800 g for 7 min. In order to exclude Kupfer cells, the HSCs were further purified by negative selection in the help of magnetic CD11b antibody beads (Miltenyi, Germany). The HSCs were then plated on plastic dishes in DMEM containing 10% fetal bovine serum (Gibco, #10099-141), 1% penicillin-streptomycin (100 mg/ml) and 4 mM L-glutamine (100 IU/ml). Cells were cultured in an incubator (37°C, 5% CO2, 95% humidity).

### Cell Culture and Transfection

Mouse primary HSCs and human LX-2 cells were cultured in endotoxin-free DMEM mixed with 10% fetal bovine serum, 1% antibiotics and 4 mM L-glutamine described above in an incubator (37°C, 5% CO2, 95% humidity). When the cells density reached 70% confluent in the 12-well plate, the cells were transfected with 1.5 μg/ml pAV-pri-miR-1 plasmid, 80 nM sja-miR-1 mimics (GenePhama, China), 80 nM sja-miR-1 inhibitor (GenePhama, China), 80 nM *Sfrp1* siRNA (GenePhama, China) or corresponding negative controls with Lipofectamine 3000 (Life Technology, #L3000-008) according to the recommended protocol. For exosome application 10 μg/mL isolated exosomes were directly added into 12-well plates for 2 days, and then cells were harvested for further detection. 293T cells were cultured in the 24-well plate with endotoxin-free DMEM mixed with 10% fetal bovine serum. When 293T cells density reached 70% confluent, the cells were transfected with plasmids or mimics accordingly. The detailed sequences of the mimics, inhibitor and siRNA are shown in Table S2.

### Isolation of Schistosomal Egg Exosomes

Parasite eggs were obtained from the livers of infected mice at 6 weeks after infection using the previous method (Cai et al., 2013). The isolated eggs were examined under a light microscope and incubated in serum-free culture medium for 24 h. Following incubation, supernatant was collected, centrifuged at 3,000 g for 15 min to eliminate remaining eggs and pellets. Then, an exoEasy Maxi Kit (QIAGEN, #76064) was applied to isolate egg exosomes according to the recommended protocol. The final purified exosomes were resuspended in 50 μl sterile PBS and kept in −80°C refrigerator for further study.

### Uptake of Schistosomal Egg Exosomes by HSCs

To investigate if the isolated egg exosomes can be internalized by the host HSCs, the egg exosomes were stained with the fluorescent lipid dye PKH67 (Sigma-Aldrich, #MINI67-1KT) according to a previous protocol with minor modification (Lässer et al., 2011). Briefly, 20 μg of the PKH67-labeled exosomes were washed and concentrated using 100 kDa ultra centrifugal filters (Millipore, #UFC810024) in order to remove redundant lipid dye. The HSCs were seeded in a 96-well plate for 6 h and the labeled exosomes were then added to the HSCs culture medium and incubated together for 1 h. At the same time, equal volume of PBS was labeled with PKH67 and added to the HSCs in a parallel experiment to exclude the non-specific labeling. Following 1 h incubation, removed the culture medium and washed the HSCs twice with PBS. Then, fixed the cells in 4% paraformaldehyde for 20 min and washed them twice with PBS. The cell nucleuses were stained using 4′6-diamidino-2-phenylindole (DAPI; Beyotime Biotechnology, China, #C1005). Finally, whether the isolated egg exosomes can be internalized by the host HSCs was observed using confocal fluorescence microscope (Zeiss, Germany).

### Pathologic Features of the Liver

After mice were anesthetized, the right liver lobes were isolated and fixed with 4% paraformaldehyde. The processed liver sections were then subjected to hematoxylin-eosin (HE) and Masson's trichrome staining to evaluate the granuloma size and hepatic fibrosis severity, which can be quantified by measuring the fibrosis grade of liver tissue as previously reported (Chiaramonte et al., 1999). For immunohistochemical staining, the liver sections were incubated with primary antibody against α-SMA (CST, #19245). Five different fields on each section were randomly selected to determine the α-SMA expression degree, the examination of which were blindly performed by two independent researchers.

### Hydroxyprolin Content Analysis

A Hydroxyproline Content Assay Kit was used to detect the hydroxyproline content of the liver tissues according to the kit's instruction (Nanjing Jiancheng, China, #A030-2). The final data were shown as Hyp (μg) /liver weight (g).

### Western Blot

Culture cells or freshly isolated cells were rinsed with pre-cooled PBS three times. Total cell protein extraction was performed using RIPA lysis buffer supplemented with protease and phosphatase inhibitors, while a Nuclear and Cytoplasmic Protein Extraction Kit (Beyotime Biotechnology, China, #P0027) was applied to extract nuclear and cytoplasmic protein. Then the protein was quantified by the BCA method and boiled at 100°C for 5 min. 30–60 μg total protein per lane was added to the 12% sodium dodecyl sulfate polyacrylamide gel electrophoresis (SDS-PAGE). Once the electrophoresis was finished, the protein was then transferred to a nitrocellulose membrane (PALL, #P-N66485) according to the wet-transferring method, and non-specific binding sites were sealed for 2 h with 5% skimmed milk powder at room temperature with shaking. Blots were probed with primary antibodies, including anti-SFRP1 (Sigma Aldrich, #AV09053), anti-β-catenin (CST, #8480), anti-GAPDH (Abcam, #ab181602) and anti-Histone H3 (CST, #4499), overnight at 4°C. After that, the membrane was washed 3 times with PBS, followed by incubation with secondary antibody for 2 h at room temperature. The rabbit anti-GAPDH antibody and anti-Histone H3 antibody (CST, #4499) were treated as internal references, while IRDye goat anti-IgG antibody (LI-COR, #926-32211) was treated as secondary antibody. After the membrane was washed 3 times with PBS, the band densities were analyzed.

### 3′UTR Luciferase Reporter Constructs

According to the online bioinformatic analysis software (miRDB), the seed sequence of sja-miR-1 was predicted to be complementary with the 3'UTR of *Sfrp1*. To detect if *Sfrp1* was a target of sja-miR-1, wild-type or mutant 3'UTRs of *Sfrp1* were chemically synthesized (Genomics) and then cloned into the pmirGLO luciferase plasmid (Promega, #E1330). The 293T cells were seeded in a 24-well plate (3 × 10^5^ cells/well). When the cells density reached up to 70%, 40 nM sja-miR-1 mimics or negative control (NC) mimics, together with 0.5 μg wild-type *Sfrp1* 3'UTR plasmid or mutant *Sfrp1* 3'UTR plasmid, were transfected into the 293T cells using lipofectamine 3000. Subsequently, the cells were cultured for 24 h and then collected. A Dual-luciferase Reporter Assay Kit (Promega, #E1901) was used to detect the effect of sja-miR-1 on the luciferase activity of the *Sfrp1* 3'UTR plasmid.

### Construction of rAAV8 Vectors

The coding sequence of sja-pri-miR-1 was obtained by PCR using the genome of adult worms as template. The PCR fragment was digested with restriction enzyme AscI and MluI and cloned between the appropriate restriction sites of the pAV-CMV-eGFP plasmid, resulting in the pAV-pri-miR-1 plasmid. To explore the possible effect of sja-miR-1 in *vivo*, the anti-sja-miR-1 sponge sequence (GACCATACTTGCGACATTCCA) was designed, synthesized and cloned into the pAV-CMV-eGFP plasmid according to a previous study (Ebert et al., 2007). Both the constructed plasmids were verified by DNA sequencing. The AAV8 vectors which were involved in this research were produced and tittered by a professional company (Vigene, China).

### AAV8 Vector Injection

For over-expression of sja-miR-1 in mice, 200 μl of rAAV8-pri-miR-1 vectors in PBS, at dose of 2 × 10^11^ particles/animal, were injected by tail vein into BALB/c mice. Similarly, rAAV8-anti-miR-1 vectors with titer of 10^12^ genome copies were given through the tail vein into mice infected percutaneously with *S. japonicum* cercariae. Equal rAAV8-SCR vectors and PBS were used as controls in this study.

### Statistical Analysis

Data were presented as Means ± SD. All results were analyzed using the GraphPad Prism 7.0 software. Student's *t*-test was applied to assess the differences between two groups, and one-way ANOVA followed by Tukey post-test was used among three or more groups. When *p* < 0.05, differences were considered to indicate statistical significance.

## Results

### Sja-miR-1 Activates HSCs *in vitro*

**Figure 1:**
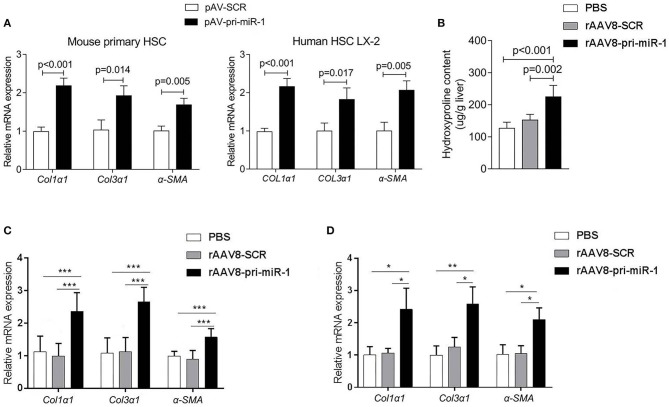
Sja-miR-1 activates HSCs and promotes the hepatic fibrosis in naive mice. **(A)** Mouse primary HSCs or LX-2 cells were transfected with pAV-pri-miR-1 plasmid and cultivated for 48 h. The level of *Col1*α*1, Col3*α*1*, and α*-Sma* were detected using qPCR (*n* = 3). **(B,C)** Mice were injected with the rAAV8-pri-miR-1 vector or rAAV8-SCR (negative control) at a dose of 2 × 10^11^ genomes or PBS via the tail vein, and liver tissues were collected at day 50 post-infection (*n* = 6). The extent of hepatic fibrosis was measured based on the hydroxyproline content **(B)** or the expression of fibrosis-related genes **(C)**. **(D)** The expression of *Col1*α*1, Col3*α*1*, and α*-Sma* in primary HSCs isolated from mice injected with various vectors at day 50 after injection were evaluated (*n* = 3). Data are presented as the Mean ± SD from three independent experiments, *p < 0.05, **p < 0.01, ***p < 0.001.

The HSCs activation represents a key feature of hepatic fibrosis and is characterized by elevated expression of the genes encoding α-SMA and collagens (Seki and Schwabe, 2015). Once liver injury occurs, quiescent HSCs undergo activation to become the major source of myofibroblasts, which highly express α-SMA and collagens (Kisseleva et al., 2012). α-SMA is regarded as a marker of HSCs activation, while accumulated collagens, especially COL1α1 and COL3α1, contribute to excessive ECM deposition (Böttcher and Pinzani, 2017). The physical properties of ECM, in addition to their chemical composition, affect HSCs activation (Friedman, 2010). To investigate the effect of sja-miR-1 on HSCs activation, we first prepared a construct expressing sja-pri-miR-1 (designated as “pAV-pri-miR-1”) (Figure S1A). To determine whether the schistosome sja-pri-miR-1 can be processed into mature sja-miR-1 in mammal cells, we cloned three perfect sja-miR-1 complementary binding sites into pmirGLO luciferase plasmid to generate a sja-miR-1 sensor plasmid (designated as “pmirGLO-miR-1”), which could regulate the expression of genes encoding firefly luciferase (*Fluc*) and renilla luciferase (*Rluc*) reporters (Figure S1A). The two constructs were transiently co-transfected into 293T cells, and the firefly luciferase activity was normalized to renilla luciferase activity. The dual-luciferase reporter assay demonstrated that the presence of the plasmid expressing Sja-pri-miR-1 resulted in the decrease of *Fluc* activity, indicating that the mature sja-miR-1 was generated in 293T cells and this miRNA suppressed expression of the *Fluc* through the sites of this miRNA (Figure S1B). Next, we transfected the Sja-pri-miR-1 expression plasmid into primary mouse HSCs and LX-2 cells respectively, and sja-miR-1 significantly increased the expression of α*-Sma Col1*α*1* and *Col3*α*1* mRNA in both cells, which illustrated that sja-miR-1 promoted activation of HSCs *in vitro* (Figure 1A).

### Sja-miR-1 Promotes Hepatic Fibrosis in Naive Mice

To investigate if sja-miR-1 can promote hepatic fibrosis in mice, we constructed recombinant adeno-associated viral serotype 8 (rAAV8) vectors to express pri-sja-miR-1 or scramble miRNA (SCR control) under the control of CMV promoter. AAV8 has been shown to transduce up to 100% liver when injected intravenously in mice (Nakai et al., 2015). Adult BALB/c male mice were injected with the vectors or PBS through the tail vein. The hydroxyproline content and expression of fibrosis-related mRNAs (*Col1*α*1, Col3*α*1*, α*-Sma*) were significantly elevated in mice injected with rAAV8-pri-miR-1 vector compared with SCR control or PBS at day 50 post injection (Figures 1B,C). This result was also validated by extension of observation time at day 100 after injection (Figures 1C,D). Moreover, the primary HSCs were isolated to measure *Col1*α*1, Col3*α*1*, and α*-Sma* expression. We found elevated expression of all the genes in mice injected with rAAV8-pri-miR-1 compared with the two control groups (Figure 1D). All these data indicated that sja-miR-1 modulated the expression of fibrosis-related genes and promoted liver fibrogenesis *in vivo*.

### Sja-miR-1 Contributes to Schistosomiasis Hepatic Fibrosis

**Figure 2:**
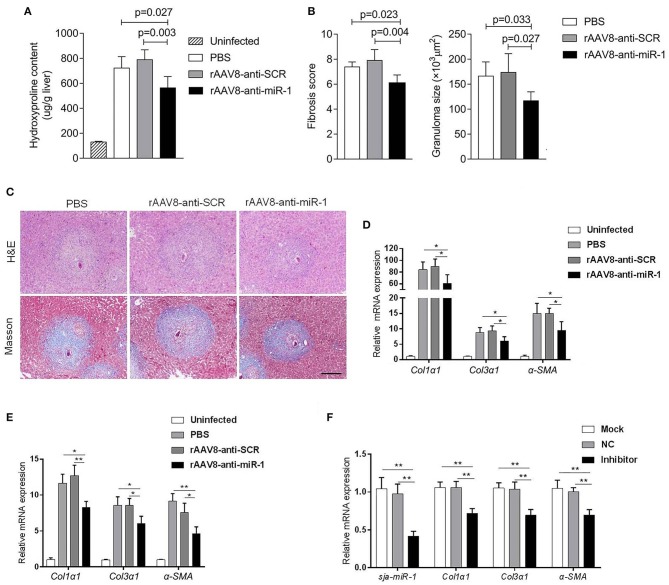
Knockdown of sja-miR-1 alleviates schistosomiasis hepatic fibrosis. **(A–E)** Mice were infected percutaneously with 16 *S. japonicum* cercariae at day 0 or remained uninfected. The infected mice received a tail vein-injection of rAAV8-anti-Sja-miR-1-sponge or rAAV8-SCR vectors at a dose of 1 × 10^12^ virus genomes or PBS via the tail vein. Liver samples were collected at day 56 after the parasite infection (*n* = 6). **(A)** Hydroxyproline content in the liver. **(B)** Fibrosis scores and granuloma size were measured from Masson's trichrome and H&E staining of liver sections. **(C)** H&E staining and Masson's trichrome staining of liver sections. Scale bar, 250 μm. **(D)** The expression of *Col1*α*1, Col3*α*1*, and α*-Sma* in the liver were detected. **(E)** The expression of *Col1*α*1, Col3*α*1*, and α*-Sma* in primary HSCs isolated from infected livers were detected (*n* = 3). **(F)** Primary HSCs were isolated from the infected mice with the parasite and then transfected with sja-miR-1 inhibitor, negative control (NC) inhibitor or transfection reagent only (Mock) for 48 h. The level of sja-miR-1 and *Col1*α*1, Col3*α*1*, and α*-Sma* were analyzed (*n* = 3). Data are presented as the Mean ± SD from three independent experiments, *p < 0.05, **p < 0.01.

**Figure 3:**
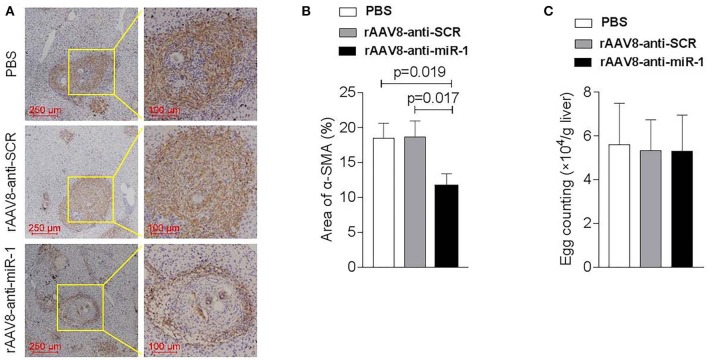
Down-regulation of sja-miR-1 partially blocked the activation of HSCs in schistosomiasis-induced liver fibrosis. Mice were infected percutaneously with 16 *S. japonicum* cercariae at day 0 or remained uninfected. rAAV8-anti-miR-1 vectors or rAAV8-SCR with titer of 10^12^ genome copies or PBS were given through the tail vein into the infected mice (*n* = 3). Liver samples were collected at 56 days post-infection. **(A)** The expression of HSC marker (α-SMA) in the livers was detected by immunohistochemical staining. **(B)** The ratio of the α-SMA-stained area to the total area was quantified. **(C)** Quantification of *S. japonicum* eggs in the liver. Data are presented as the Mean ± SD from three independent experiments.

To evaluate if sja-miR-1 was involved in the parasite-induced liver fibrosis during the parasite infection, we generated a rAAV8-anti-miR-1-sponge vector for knockdown of sja-miR-1 according to previous study (Ebert et al., 2007) (Figure S2A). The 293T cells were co-transfected with sja-miR-1 sensor plasmid, sja-miR-1 expression plasmid and sponge vector to examine whether the sponge vector could inhibit sja-miR-1 function. The dual-luciferase reporter assay showed that the sponge vector specifically inhibited sja-miR-1 function in the 293T cells (Figure S2B). BALB/c mice were infected with the parasite cercariae, followed by intravenously injection with the vectors or PBS at 10 days after infection. The liver tissues were obtained at 8 weeks after injection of the vectors. Mice injected with rAAV8-anti-miR-1 sponge vector revealed a significant reduction in ECM production compared with the control mice, characterized by hydroxyproline quantification (Figure 2A) and Masson's trichrome staining (Figures 2B,C), indicating the attenuation of hepatic fibrosis. Moreover, a significant reduction of the size of hepatic granulomas was visualized by H&E staining (Figures 2B,C). This alleviation of liver fibrosis was also validated by quantification of fibrosis-related genes revealing the expression of *Col1*α*1, Col3*α*1*, and α*-Sma* were dramatically decreased in livers of mice injected with rAAV8-anti-miR-1 sponge vector compared with the controls (Figure 2D). Moreover, immunohistochemical (IHC) staining showed that the sponge vector also reduced the protein level of α-SMA compared with the controls (Figures 3A,B). The rAAV8 vector, as expected, had no effect on the parasite eggs production in mice (Figure 3C).

Because of the key roles of HSCs in schistosome-induced hepatic fibrosis (Burke et al., 2009), we then obtained primary HSCs derived from the infected mice injected with the sponge vector to quantify the expression of genes related to HSCs activation. The results showed a lower level of *Col1*α*1, Col3*α*1*, and α*-Sma* in the HSCs of mice injected with rAAV8-anti-miR-1 sponge vector compared with the control mice (Figure 2E).

To further validate the pro-fibrotic effect of sja-miR-1 and its existence in HSCs of mice infected with *S. japonicum*, the primary HSCs isolated from the infected mice at 56 days post-infection were transfected with sja-miR-1 inhibitor or NC inhibitor *in vitro*. The qPCR analysis showed that the miR-1 inhibitor treatment significantly reduced the expression of the genes encoding α*-Sma, Col1*α*1*, and *Col3*α*1* compared with the NC or Mock (Figure 2F). These results suggested that the parasite-derived sja-miR-1 may be a virulence factor contributing to pathogenicity of schistosomiasis.

### *S. japonicum* Egg-Derived Exosomes and Internalization of Sja-miR-1 in HSCs

Exosomes cargo, including miRNAs, play a regulatory role in host-pathogen interactions (Szempruch et al., 2016). In our previous research, we found that sja-miR-1 was abundantly present in host HSCs through RNA sequencing (He et al., 2019). To ascertain whether the *S. japonicum* egg-derived exosomes can enter host cells, we labeled *S. japonicum* exosomes with the lipid dye PKH67 and incubated the labeled exosomes in primary HSCs derived from mice and LX-2 cell line *in vitro*. We revealed efficient internalization of the egg exosomes by both primary HSCs and LX-2 cells using confocal image analysis (Figure S3A). To investigate the roles of *S. japonicum* egg exosomes in activating HSCs, we incubated them with primary HSCs from naive mice for 24 h and found that sja-miR-1 was detectable in the cells treated with the exosomes (Figure S3B). Crucially, the exosomes promoted activation of HSCs, evidenced by elevated level of *Col1*α*1, Col3*α*1*, and α*-Sma*. While pretreatment with sja-miR-1 inhibitor abrogated the effects of exosomes on level of *Col1*α*1, Col3*α*1*, and α*-Sma* production compared with the controls (Figure S3C). These data suggested that sja-miR-1 carried by the egg-derived exosomes could be transferred to the HSCs and regulated fibrosis-related genes in HSCs.

### *Sfrp1* Is a Target Gene of Sja-miR-1

**Figure 4:**
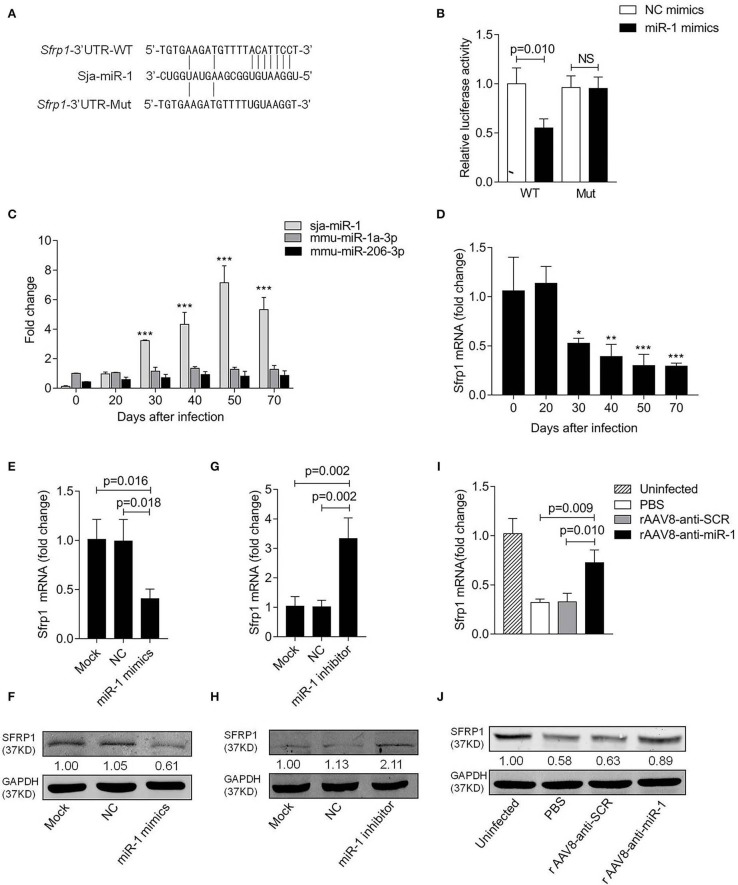
*Sfrp1* is the direct target gene of sja-miR-1. **(A)** Sequence alignment of sja-miR-1 and the target region in the 3'UTR of *Sfrp1*. The corresponding luciferase reporter constructs were designated as pmirGLO-*Sfrp1*-3'UTR-WT and pmirGLO-*Sfrp1*-3'UTR-Mut, respectively. **(B)** Luciferase reporter assays of 293T cells transfected with pmirGLO-*Sfrp1*-3'UTR-WT or pmirGLO-*Sfrp1*-3'UTR-Mut in the absence or presence of sja-miR-1 mimics (*n* = 3). WT, pmirGLO-*Sfrp1*-3'UTR-WT; Mut, pmirGLO-*Sfrp1*-3'UTR-Mut. **(C,D)** The expression of sja-miR-1, mmu-miR-1a-3p, mmu-miR-206-3p and *Sfrp1* in primary HSCs isolated from the infected mice at various time points post-infection. **(E,F)** Primary HSCs isolated from naive mice were transfected with the sja-miR-1 mimics, a negative control (NC) mimics or transfection reagent only (Mock) for 48 h. The expression of SFRP1 was detected by qPCR or western blot (*n* = 3). **(G,H)** Primary HSCs isolated from infected mice were transfected with sja-miR-1 inhibitor, a negative control (NC) inhibitor or transfection reagent only (Mock) for 48 h. The expression of SFRP1 was detected by qPCR or western blot (*n* = 3). **(I,J)** The expression of SFRP1 in primary HSCs from the infected mice treated with the sponge vectors at day 56 post-infection was detected using qPCR or western blot (*n* = 3). Data are presented as the Mean ± SD from three independent experiments, *p < 0.05, **p < 0.01, ***p < 0.001. NS, not significant.

The online bioinformatic analysis using miRDB software suggested the *Sfrp1* as the potential target gene of sja-miR-1. To further validate the target gene, the luciferase reporter constructs were generated that contained 3'UTR of *Sfrp1* of either wild type (*Sfrp1*-3'UTR-WT) or mutant (*Sfrp1*-3'UTR-Mut) complementary sites of sja-miR-1 (Figure 4A). The luciferase assay indicated a significant decrease in the luciferase activity in the cells transfected with *Sfrp1*-3'UTR-WT and sja-miR-1 mimics compared with the control groups (Figure 4B). We then analyzed the expression of sja-miR-1 and *Sfrp1* in HSCs isolated from the liver samples of infected mice at various time points post infection. The qPCR analysis showed that sja-miR-1 level was significantly up-regulated since day 30 and peaked at day 50 (Figure 4C). In contrast, *Sfrp1* mRNA in HSCs was significantly decreased since post infection, which was reversely related to the sja-miR-1 level in HSCs (Figure 4D). Next, we detected both mRNA and protein levels of SFRP1 in primary HSCs transfected with sja-miR-1 mimics *in vitro*. The results showed that both mRNA and protein level of *Sfrp1* were dramatically decreased in the HSCs transfected with sja-miR-1 mimics (Figures 4E,F), while inhibition of sja-miR-1 in the HSCs derived from the infected mice by the sja-miR-1 inhibitor dramatically elevated the levels of both *Sfrp1* mRNA and protein (Figures 4G,H). Furthermore, the primary HSCs isolated from the livers of infected mice treated with the sponge vector showed significant up-regulation of the *Sfrp1* expression compared with the controls (Figures 4I,J). Thus, our result suggested that *Sfrp1* could be a target gene of sja-miR-1 in HSCs.

### Sja-miR-1 Activates Wnt/β-Catenin Pathway in HSCs by Targeting *Sfrp1*

**Figure 5:**
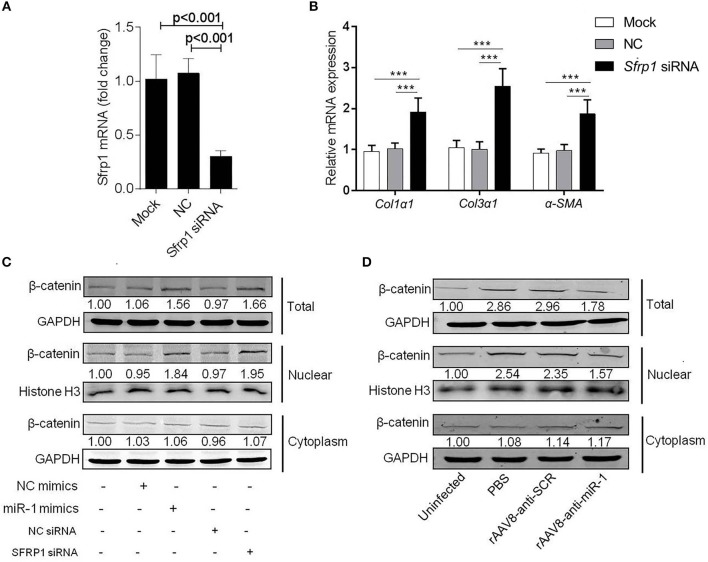
Sja-miR-1 activates HSCs via Wnt/β-catenin signaling pathway. **(A,B)** Primary HSCs isolated from naive mice were transfected with *Sfrp1* siRNA, a negative control siRNA (NC) or transfection reagent only (Mock) for 48 h. The expression of *Sfrp1* **(A)**, *Col1*α*1, Col3*α*1*, and α*-Sma*. **(B)** were detected (*n* = 3). **(C)** The protein level of total, nuclear, and cytoplasm β-catenin in HSCs with various treatments indicated were detected using western blot. **(D)** The protein level of β-catenin in primary HSCs isolated from infected mice treated with sponge vectors were detected. Data are presented as the Mean ± SD from three independent experiments, ***p < 0.001.

It was reported that SFRP1 can inhibit Wnt signaling pathway, which was involved in promoting hepatic fibrosis by enhancing HSCs activation and survival (Myung et al., 2007). To investigate the biological functions of SFRP1 in the HSC activation, the primary HSCs from naive mice were transfected with *Sfrp1* siRNA and qPCR analysis showed that *Sfrp1* siRNA reduced 70% level of *Sfrp1* (Figure 5A), which led to the activation of HSCs, evidenced by significant elevation of *Col1*α*1, Col3*α*1*, and α*-Sma* compared with the controls (Figure 5B).

To investigate involvement of the Wnt/β-catenin signaling in the sja-miR-1-mediated activation of HSCs, the protein level of β-catenin was detected in HSCs treated with sja-miR-1 mimics or *Sfrp1* siRNA. Consistent with previous reports that SFRP1 functioned as a negative modulator of Wnt/β-catenin pathway (Sklepkiewicz et al., 2015), western blot analysis showed that the nuclear protein level of β-catenin was increased in the HSCs transfected with sja-miR-1 mimics or *Sfrp1*siRNA compared with the controls, whereas no significant difference was detected in the cytoplasm protein level of β-catenin (Figure 5C). Next, we analyzed β-catenin level in primary HSCs isolated from mice treated with the sponge vector and showed that β-catenin protein was obviously decreased in total HSCs homogenate and the nuclear protein compared with the control vector (Figure 5D). These data indicated that sustained and efficient suppression of sja-miR-1 led to marked elevation of SFRP1 and thereby inhibited the Wnt/β-catenin signaling pathway and collagens production.

## Discussion

Sja-miR-1 was abundant in *Schistosoma japonicum* and considered playing an important role in schistosome sexual maturation (Cai et al., 2013; Han et al., 2015). This study provides the following evidence that the sja-miR-1 is involved in the occurrence and progression of hepatic fibrosis during schistosome infection: (1) sja-miR-1 was present in HSCs and involved in host HSC activation by targeting the *Sfrp1*; (2) rAAV8-mediated delivery of sja-miR-1 to the mouse liver promotes hepatic fibrosis *in vivo*; (3) the sustained inhibition of sja-miR-1 alleviated schistosomiasis hepatic fibrosis via reduction in production of collagens and α-SMA in the infected mice with the parasite; (4) the sja-miR-1 inhibitor reduced the expression of fibrosis-related genes in the HSCs isolated from infected mice or in the egg exosome-activated HSCs *in vitro*.

Wnt proteins are involved in the modeling and remodeling processes (van Gijn et al., 2002; Moon et al., 2004; Reya and Clevers, 2005; Brade et al., 2006). Wnt proteins are divided into two classes, involving the canonical and non-canonical Wnt pathways. The former stabilizes intracellular β-catenin, which can transfer β-catenin into the nucleus to activate the Wnt-dependent genes via regulating the relationship between the transcription factors and the transcriptional co-repressors (Huelsken and Behrens, 2002; Moon et al., 2004). Altered Wnt signaling is related to various disease progression including liver diseases (Thompson and Monga, 2007). Accumulated studies reveal that the activation of Wnt/β-catenin signaling can promote hepatic fibrosis by enhancing HSCs activation via HSCs proliferation, and ECM accumulation and also play an important role in schistosomiasis-induced hepatic fibrosis (Jiang et al., 2006; Myung et al., 2007). SFRP1 is homologous protein of the extracellular cysteine-rich-domain of the Wnt receptor Frizzled, but lacks the transmembrane and intracellular domain (Yang et al., 2009). SFRP1 protein functions as a negative regulator of Wnt/β-catenin signaling via inhibiting the binding of the Wnt receptor and thereby suppressing ligand-receptor interactions and signal transduction (Jones and Jomary, 2002; Kang et al., 2014). The reduction in production of SFRP1 leads to the activation of Wnt/β-catenin signaling pathways (Sklepkiewicz et al., 2015). It was demonstrated that the expression of fibrosis-related markers was elevated in SFRP1 knock-out mice (Matsuyama et al., 2014; Sklepkiewicz et al., 2015). Here, our study revealed that *Sfrp1* gene was a direct target gene of sja-miR-1 both *in vitro* and *in vivo* models, and the sja-miR-1 mediated down-regulation of *Sfrp1* elevated the expression of collagens and α-SMA and thereby activated the HSCs. Further, sustained and efficient inhibition of sja-miR-1 *in vivo* led to significant increase in the expression of SFRP1 and reduction in the collagen production. In addition, siRNA-mediated inhibition of *Sfrp1* expression remarkably increased intra-nuclear β-catenin level, and consequently caused hepatic fibrosis. Therefore, these findings demonstrate that SFRP1 is a negative regulator of Wnt/β-catenin pathway which is consistent with the previous findings (Jones and Jomary, 2002; Kang et al., 2014).

The hepatic fibrosis is main pathology of schistosomiasis. The previous studies demonstrated the Th2 cytokines such as TGF-β1 and IL-13 were major profibrogenic factors. Recently, we reported that the sja-miR-2162 modulated pathogenicity of schistosomiasis in the infected mice by targeting the transforming growth factor beta receptor III (He et al., 2019). In this study, we demonstrated that sja-miR-1 derived from the parasite also contributed to the schistosomiasis hepatic fibrosis by regulating the fibrosis-related genes in a cross-species manner, which enriched our understanding of the pathogenesis of schistosomiasis. Further, the miRNAs derived from parasites may represent the emerging generic regulators that are involved in the pathogenesis of other parasite diseases, and the rAAV8-mediated sustained suppression of the heterogenous miRNAs may present a promising intervention for the disease therapy.

## Data Availability Statement

All datasets generated for this study are included in the article/Supplementary Material.

## Ethics Statement

The animal study was reviewed and approved by Animal Ethics Committee of Naval Medical University (approval number FYXK [Shanghai] 2014-0003).

## Author Contributions

YW, XF, and WP designed research and wrote the paper. YW, XF, NL, XW, and XL performed research. XH, YW, DZ, and WP analyzed data. All authors read and approved the final manuscript.

### Conflict of Interest

The authors declare that the research was conducted in the absence of any commercial or financial relationships that could be construed as a potential conflict of interest.
