# Supplementary material for: A MicroRNA Derived From Schistosoma japonicum Promotes Schistosomiasis Hepatic Fibrosis by Targeting Host Secreted Frizzled-Related Protein 1
Source: Front Cell Infect Microbiol. 2020 Mar 13;10:101. doi: 10.3389/fcimb.2020.00101 (PMC7082693; doi:10.3389/fcimb.2020.00101)
Supplement: Supplementary file 1 [file Presentation_1.PPT]

## Slide 1
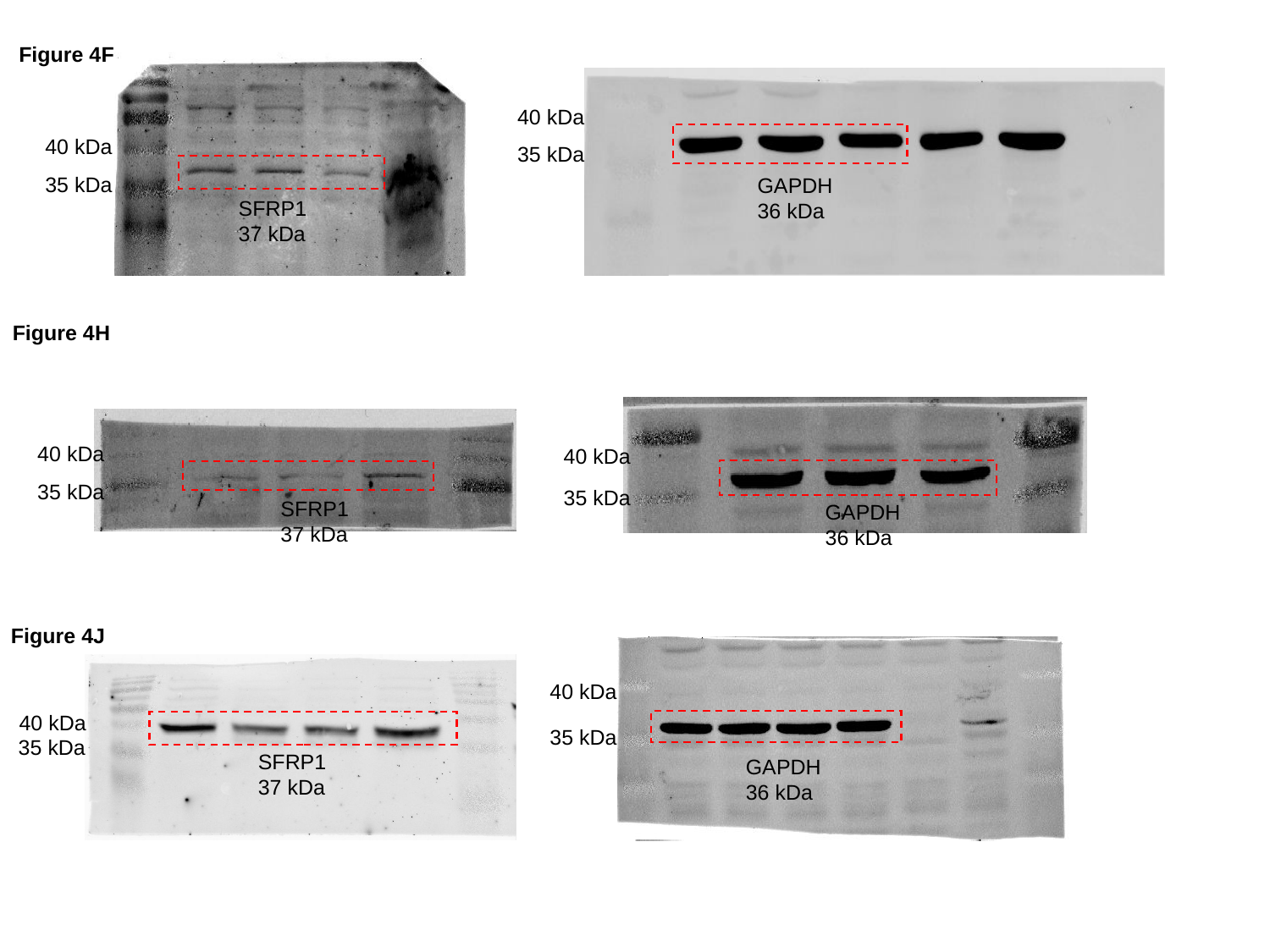

Figure 4F
40 kDa
35 kDa
SFRP1 37 kDa
40 kDa
35 kDa
GAPDH 36 kDa
Figure 4H
40 kDa
35 kDa
GAPDH 36 kDa
40 kDa
35 kDa
SFRP1 37 kDa
Figure 4J
40 kDa
35 kDa
GAPDH 36 kDa
40 kDa
35 kDa
SFRP1 37 kDa

## Slide 2
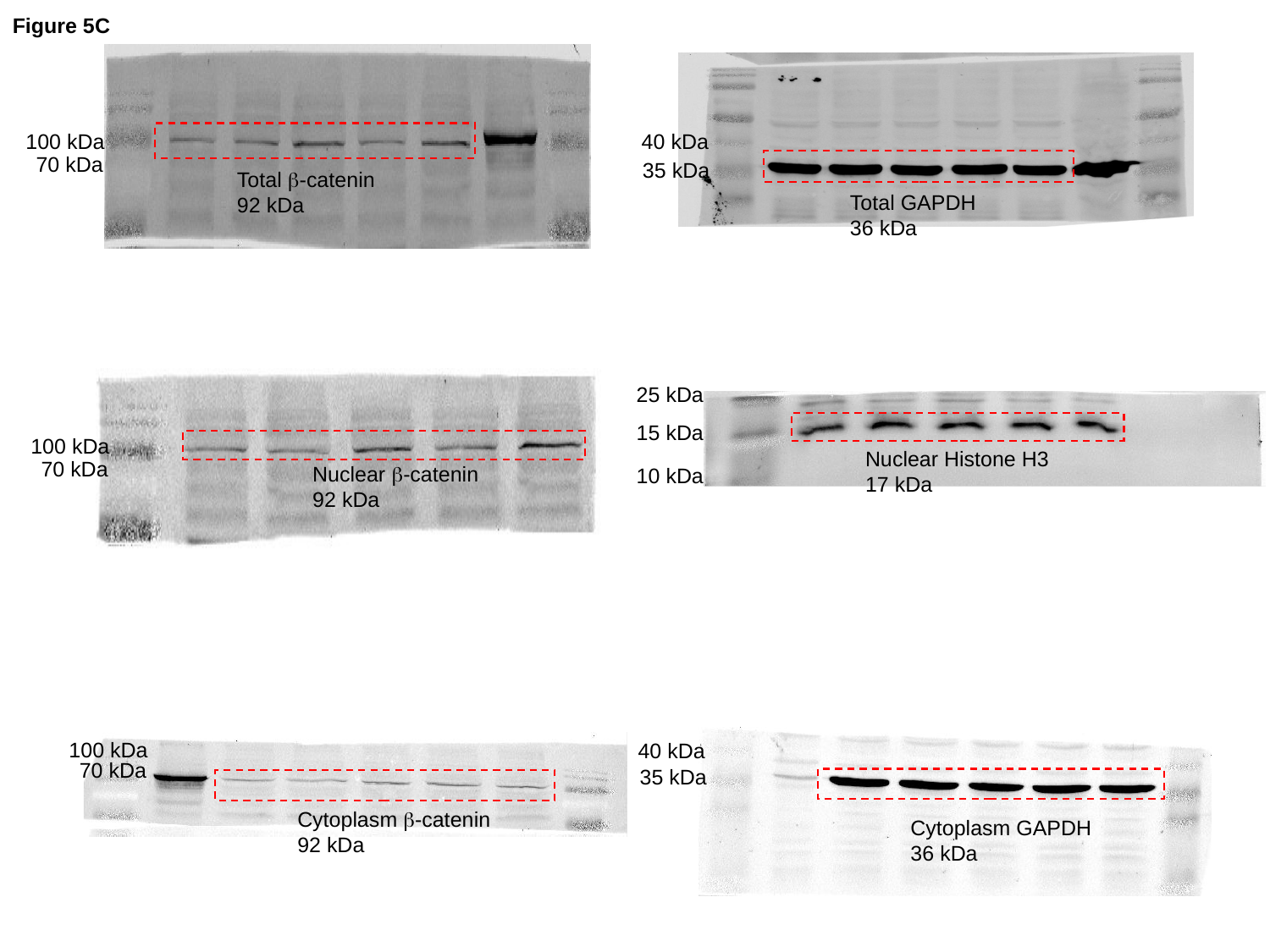

Figure 5C
100 kDa
70 kDa
Total β-catenin
92 kDa
40 kDa
35 kDa
Total GAPDH 36 kDa
25 kDa
15 kDa
Nuclear Histone H3
17 kDa
10 kDa
100 kDa
70 kDa
Nuclear β-catenin
92 kDa
40 kDa
35 kDa
Cytoplasm GAPDH 36 kDa
100 kDa
70 kDa
Cytoplasm β-catenin 92 kDa

## Slide 3
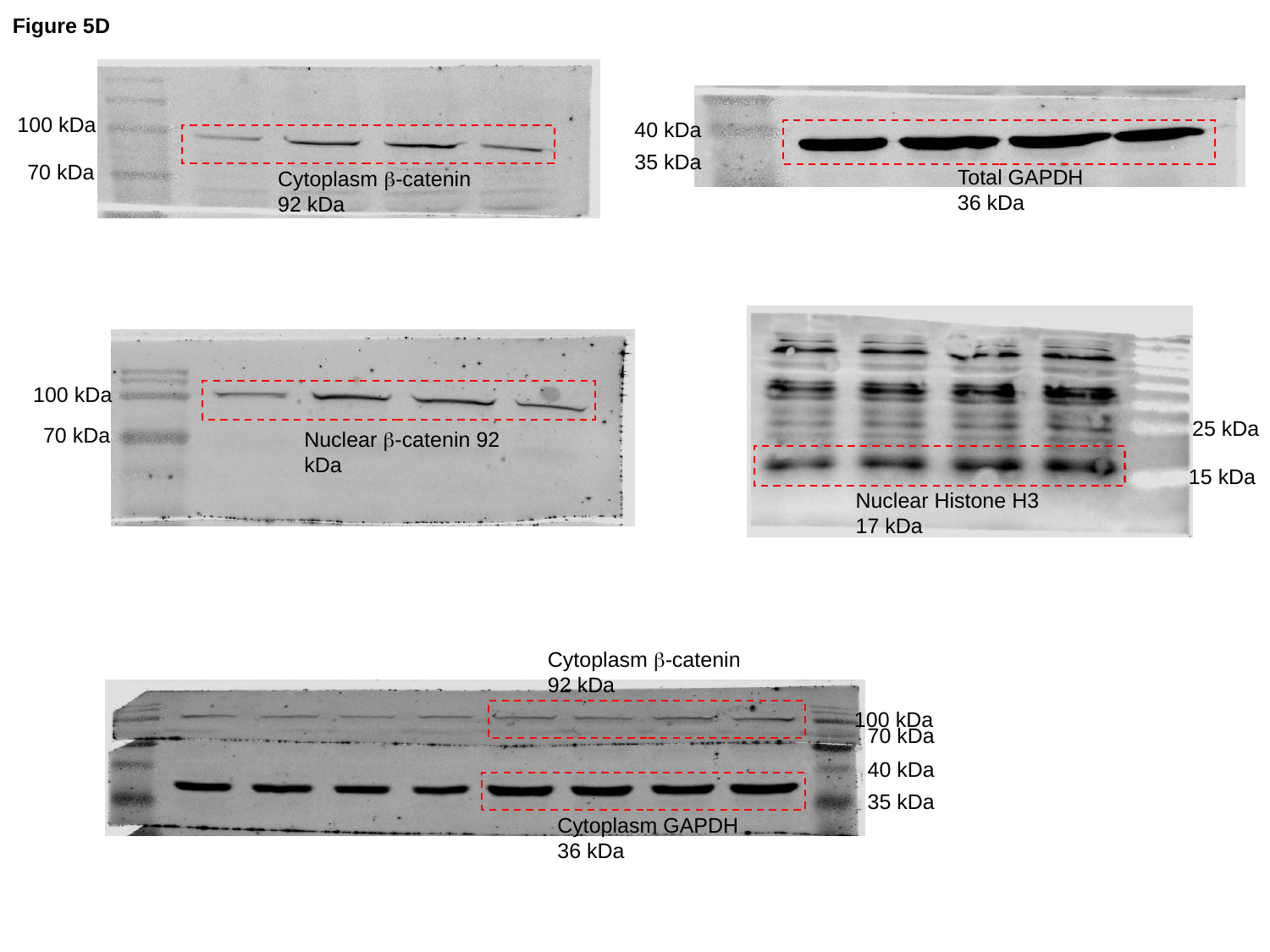

Figure 5D
100 kDa
70 kDa
Cytoplasm β-catenin 92 kDa
40 kDa
35 kDa
Total GAPDH 36 kDa
25 kDa
15 kDa
Nuclear Histone H3
17 kDa
100 kDa
70 kDa
Nuclear β-catenin 92 kDa
Cytoplasm β-catenin 92 kDa
100 kDa
70 kDa
40 kDa
35 kDa
Cytoplasm GAPDH 36 kDa
