# Supplementary material for: A MicroRNA Derived From Schistosoma japonicum Promotes Schistosomiasis Hepatic Fibrosis by Targeting Host Secreted Frizzled-Related Protein 1
Source: Front Cell Infect Microbiol. 2020 Mar 13;10:101. doi: 10.3389/fcimb.2020.00101 (PMC7082693; doi:10.3389/fcimb.2020.00101)

**Involvement of A** ***Schistosoma japonicum* MicroRNA in Promoting Schistosomiasis Hepatic Fibrosis by Targeting Host** **Secreted Frizzled-Related Protein 1**

**Authors**

Yange Wang^1†^, Xiaobin Fan^1†^, Nanhang Lei^1†^, Xing He^1^, Xiaoxi Wang^1^, Xufeng Luo^1^, Dongmei Zhang^1*^, Weiqing Pan^1*^

**Authors’ Affiliations**

^1^ Department of Tropical Diseases, Naval Medical University, Shanghai, China.

***Correspondence:**

Weiqing Pan

Email: [wqpan0912@aliyun.com](mailto:wqpan0912@aliyun.com)

Dongmei Zhang

Email: [dmzhangcn@163.com](mailto:dmzhangcn@163.com)

**Supplementary Materials**

**Table S1.** **Sequences of primers used for qPCR.**

| **Gene** | **Name** | **Sequence (5’-3’)** |
| --- | --- | --- |
| sja-miR-1 | Reverse transcription stem-loop primer | GTCGTATCCAGTGCAGGGTCCGAGGTATTCGCACTGGATACGACGACCAT |
|  | Forward primer | ATGGTTCGTGGGTGGAATGTGG |
|  | Reward primer | GCAGGGTCCGAGGTATTC |
| mmu-U6 | Forward primer | GCTTCGGCAGCACATATACTAAAAT |
|  | Reward primer | CGCTTCACGAATTTGCGTGTCAT |
| Mouse *Col1α1* | Forward primer | GCACGAGTCA CACCGGAAC |
|  | Reward primer | CCAATGTCCAAGGGAGCCAC |
| Mouse *Col3α1* | Forward primer | TGGTCCTCAGGGTGTAAAGG |
|  | Reward primer | GTCCAGCATCACCTTTTGGT |
| Mouse *α-Sma* | Forward primer | GTCCCAGACATCAGGGAGTAA |
|  | Reward primer | TCGGATACTTCAGCGTCAGGA |
| Mouse *β-Actin* | Forward primer | GGCTGTATTCCCCTCCATCG |
|  | Reward primer | CCAGTTGGTAACAATGCCATGT |
| Mouse *Sfrp1* | Forward primer | TACTGGCCCGAGATGCTCAA |
|  | Reward primer | GAGGCTTCCGTGGTATTGGG |
| Human *COL1α1* | Forward primer | GAGGGCCAAGACGAAGACATC |
|  | Reward primer | CAGATCACGTCATCGCACAAC |
| Human *COL3α1* | Forward primer | GGAGCTGGCTACTTCTCGC |
|  | Reward primer | GGGAACATCCTCCTTCAACAG |
| Human *α-SMA* | Forward primer | AAAAGACAGCTACGTGGGTGA |
|  | Reward primer | GCCATGTTCTATCGGGTACTTC |
| Human *GAPDH* | Forward primer | AACGACCCCTTCATTGAC |
|  | Reward primer | TCCACGACATACTCAGCAC |

**Table S2.** **Sequences of mimics, inhibitor and siRNA.**

| **Name** | **Sequence (5’-3’)** |
| --- | --- |
| sja-miR-1 mimics | Sense: UGGAAUGUGGCGAAGUAUGGUC  Anti-sense: CCAUACUUCGCCACAUUCCAUU |
| NC mimics  sja-miR-1 inhibitor  NC inhibitor | Sense: UUCUCCGAACGUGUCACGUTT  Anti-sense: ACGUGACACGUUCGGAGAATT GACCAUACUUCGCCACAUUCCA  UCUACUCUUUCUAGGAGGUUGUGA |
| *Sfrp1* siRNA | Sense: AACUUCUUGGGGACAAUCUUC  Anti-sense: AAGAAGAUUGTCCCCAAGAAG |

**Figure S1. The pAV-pri-miR-1 plasmid can be successfully processed into mature sja-miR-1 in mammalian cells.** **(A)** Schematic diagram of pAV-pri-miR-1 plasmid and sja-miR-1 sensor plasmid used in this study. **(B)** The pAV-pri-miR-1 plasmid was co-transfected with miR-1 sensor plasmid into 293T cells for 24 h. The ratio of *Fluc*/*Rluc* activity reflected the level of active sja-miR-1 in the transfected cells (n=3). **(C, D)** Littermate mice were injected with the rAAV8-pri-miR-1 vector or rAAV8-SCR (negative control) at a dose of 2 × 10^11^ genome copies or PBS via the tail vein, and liver tissues were collected at 100 days post-infection (n=6). The extent of hepatic fibrosis was measured based on the hydroxyproline content **(C)** or the expression of fibrosis-related genes **(D)**. Data are presented as the Mean ± SD from three independent experiments, ***p* < 0.01, ****p* < 0.001.


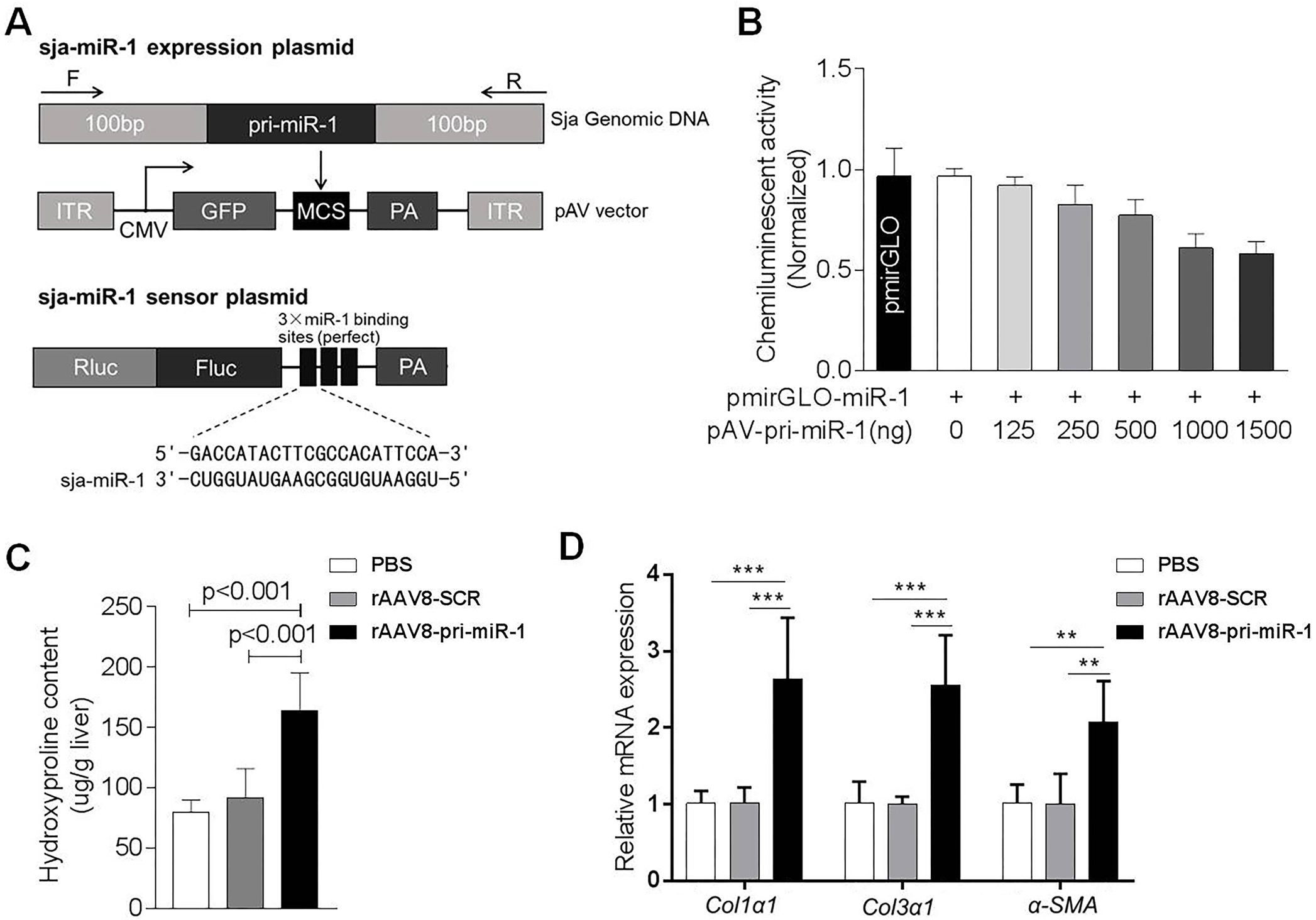


**Figure S2. The potency and specificity of anti-miR-1 sponge plasmid were verified *in vitro*.** **(A)** Schematic diagram of anti-miR-1 sponge plasmid. **(B)** 293T cells were transfected simultaneously with pAV-pri-miR-1 plasmid, miR-1 sensor plasmid and anti-miR-1 sponge plasmid for 24 h. The ratio of *Fluc*/*Rluc* activity was analyzed (n=3). Data are presented as the Mean ± SD from three independent experiments.


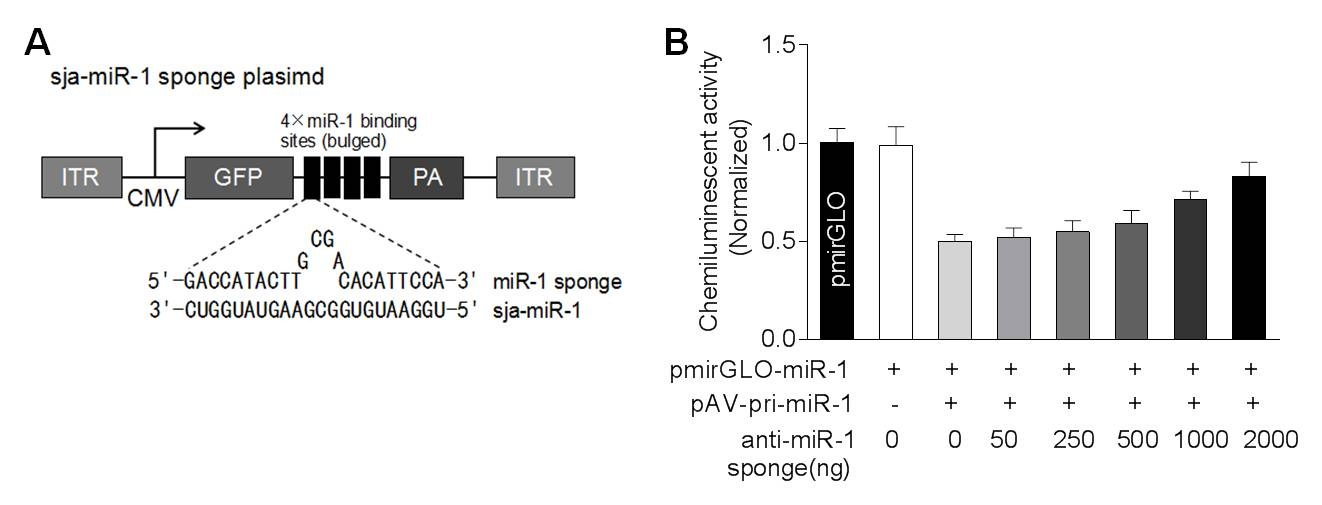


**Figure S3. *S. japonicum* egg exosomes associated sja-miR-1 promotes the activation of host HSCs.** **(A)** Uptake of *S. japonicum* egg exosomes by HSCs was detected using ﬂuorescence microscopy. PKH67-labeled *S. japonicum* egg exosomes and PHK67-PBS were incubated with LX-2 or primary mouse HSCs for 1 h. Nuclei were stained with DAPI (blue). Scale-bars, 20 μm. **(B, C)** Primary HSCs isolated from naive mice were incubated with *S. japonicum* egg exosomes, combined or not combined with transfection of sja-miR-1 inhibitor. The expression of sja-miR-1 and markers of HSCs activation were detected. Data are presented as the Mean ± SD from three independent experiments, **p* < 0.05, ***p* < 0.01, ****p* < 0.001.


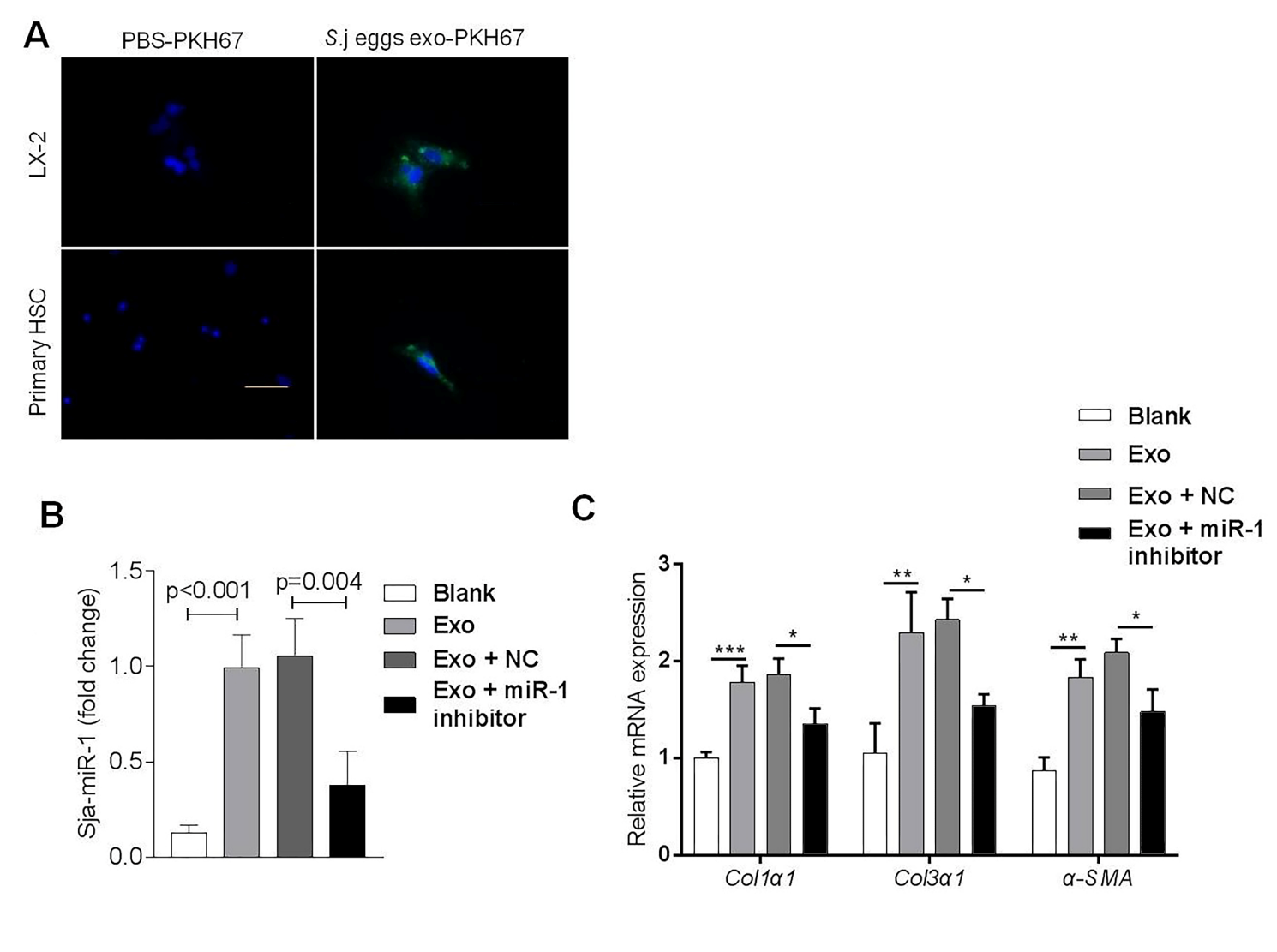

Supplement: Supplementary file 2 [file Data_Sheet_1.doc]
